# Impact of socio-economic deprivation on death rates after surgery for upper gastrointestinal tract cancer

**DOI:** 10.1038/sj.bjc.6603315

**Published:** 2006-08-22

**Authors:** Y Leigh, V Seagroatt, M Goldacre, P McCulloch

**Affiliations:** 1 Nuffield Department of Surgery, University of Oxford, John Radcliffe Hospital, Headley Way, Headington, Oxford OX3 9DU, UK; 2 Unit of Health Care Epidemiology, Department of Public Health, University of Oxford, Old Road Campus, Headington, Oxford OX3 7LF, UK

**Keywords:** oesophagectomy, gastrectomy, mortality, socio-economic deprivation

## Abstract

We hypothesised that socio-economic deprivation in England may be a prognostic factor for death after oesophagectomy or gastrectomy for cancer of the upper gastrointestinal tract. We analysed statistical data from hospital records linked to death records for patients who underwent operations for oesophageal and gastric cancer in England from April 1998 to March 2002. The patients were stratified into quintiles according to the index of multiple deprivation (IMD) (2000) for their place (ward) of residence. Age and sex standardised death rates at 30 and 90 days for each deprivation quintile were calculated. Following oesophagectomy, death rates showed a significant association with IMD. They increased with increasing levels of deprivation: the odds ratio for death, comparing highest with lowest quintile for deprivation, was 1.37 (95% confidence interval 1.03–1.85) at 30 days and 1.30 (1.04–1.64) at 90 days. Following gastrectomy, the death rates showed smaller and nonsignificant associations with IMD with odds ratios of 1.16 (0.84–1.62) and 1.10 (0.86–1.41), respectively. There is a significant association between social deprivation and death after oesophagectomy, but less of an association, if any, after gastrectomy in current UK practice.

Surgery is currently the mainstay of potentially curative treatment for oesophageal and gastric cancer. However, despite improvements in perioperative care, the operations are not without risk. Reviews of international studies concluded that postoperative death rates following oesophageal cancer operations were 8.9% in western countries (Jamieson *et al*, 2004), and that 30-day death rates following gastrectomy for cancer were 7.6% (Grossmann *et al*, 2002). Prognostic factors for postoperative hospital death after upper gastrointestinal tract (GI) cancer operations have been studied using multivariable logistic regression models comprising patient-related and hospital-related variables. Independently predictive patient-related variables include the physiological component of the POSSUM score (McCulloch *et al*, 2003), tumour stage, ASA grade and poor cardiac, hepatic or respiratory function (Bartels *et al*, 1998). Predictive hospital-related variables included choice of operation (Dimick *et al*, 2005), the annual case volumes of the hospital for oesophagectomy (Birkmeyer *et al*, 2003) and the individual surgeon's operative volume. Overall quality of care might be expected to show some prognostic benefit, and recent reports from the USA National Cancer Institute Centres of Excellence record even better outcomes in centres of excellence than in standard high volume centres for oesophagectomy (Birkmeyer *et al*, 2005), perhaps due to superior overall management of the surgical patient.

Although multivariate regression modelling shows that these factors account for some of the variance found in these studies, a substantial proportion of the variance remains unexplained. The impact of socio-economic deprivation on outcomes after upper GI cancer surgery is not well documented, and it may account for some of the unexplained variance. Socio-economic deprivation has been shown to be an independent risk factor associated with increased mortality (Taylor *et al*, 2003) and morbidity (Johnston *et al*, 2004) after coronary artery bypass surgery. Deprivation is also associated with relatively low operation rates in carotid endarterectomy surgery (Mackenzie *et al*, 2003), and with a lower survival rate after colorectal surgery (Hole and McArdle, 2002). We hypothesised that social deprivation (a measurement composed of several elements) may be a predictive factor contributing to mortality after upper GI cancer surgery, and that the death rates associated with oesophagectomy and gastrectomy in England may be higher in areas with high levels of social deprivation than in areas with low levels.

## MATERIALS AND METHODS

Anonymised statistical records of hospital admissions in England were obtained from a Hospital Episode Statistics (HES) database, linked to data from death certificates, for the period from April 1998 to March 2002. Ethical committee approval for the work programme to analyse this database had been granted by the Central and South Bristol Research Ethics Committee (Ref: 04/Q2006/176). Cases with an Office of Population and Censuses (fourth revision) operation code for either oesophagectomy or gastrectomy (OPCS codes G01–G03 excluding G02.1, and G27, G28.1–G28.3, respectively), in conjunction with a diagnosis code for oesophageal or gastric cancer (International Classification of Diseases, revision 10, codes C15.1–C16 and C16.1–C16.9) were selected as the study population. For both OPCS and ICD codes, cases were included if the relevant codes appeared in any field. No patient-identifiable data were requested by or revealed to the investigators.

The IMD (2000), a measurement tool developed initially for the Department for Transport Local Government and the Regions, was used to measure social deprivation at electoral ward level, using the postcode for each patient. Wards were ranked on their IMD score and divided in to quintiles based on their ranks, with quintile 1 representing the highest level of deprivation. Within each quintile, age and sex standardised death rates for deaths occurring within 30 or 90 days of admission were calculated by the indirect method, taking the whole study population as the standard. Confidence intervals (CI) for the standardised death rates were calculated assuming a Poisson distribution for the observed numbers of deaths.

We used logistic regression to estimate the trends in death rates across the IMD quintiles and their statistical significance. For each operation, a logistic model with a linear term (linear on a logistic scale) for the IMD quintiles were fitted to data, with adjustments for sex and age (grouped in 5-year age bands). The IMD quintiles were also fitted as categorical variables and odds ratios calculated relative to the quintile for least deprivation. To determine whether the IMD trend varied by age, the data were grouped into two broad age bands. A model was fitted with separate linear trends for each age group. If this produced a significant improvement in fit, compared with fitting the model with a common trend, then the IMD trend was judged to vary with age.

## RESULTS

There were records of 6282 patients who had an oesophagectomy and 4865 that had a gastrectomy. Patients with no data on IMD were excluded from the analysis (132 and 33, respectively). Of patients, 6150 had an oesophagectomy, of which 74% were male. In all, 4832 patients had a gastrectomy, of which 65% were male. The average age at operation was 63.9 and 69.8 for oesophagectomy and gastrectomy, respectively. Patients in the more deprived areas were, on average, younger than those in the less deprived areas (Table 1).

The overall age and sex standardised case fatality rates (CFR) following oesophagectomy were 8.3 and 13.4% at 30 and 90 days, respectively. Following gastrectomy, the 30- and 90-day death rates were 8.9 and 15.1%, respectively. The death rates at 30 and 90 days for each IMD (2000) quintile for oesophageal cancer resection admissions are shown in Table 2, with their CI. Mortality following oesophagectomy showed a significant trend (*χ*^2^=9.5, df=1, *P*=0.002 at 30 days; and 12.6, *P*=0.0004 at 90 days), with standardised death rates rising with increasing levels of social deprivation. For gastric cancer, shown in Table 3, there was no significant trend across the quintiles (*χ*^2^=0.6, df=1, *P*=0.46 at 30 days; and 2.0, *P*=0.16 at 90 days).

The odds ratios of mortality at 30 and 90 days for the most deprived quintile relative to the least deprived are shown in Table 4 for each operation, together with their 95% CIs.

Table 5 compares the sex and age-truncated standardised CFRs for the IMD quintiles dichotomised by age at 65 and 70 years for oesophagectomy and gastrectomy, respectively. These rates fluctuate somewhat, as they are based on smaller numbers than the all-ages death rates. No significant differences in the IMD trends were found between the younger and older patients for either the 30- or 90-day death rates for oestophagectomy and for gastrectomy (positive values indicates that death rates increase as deprivation increases).

## DISCUSSION

Postoperative mortality following operations for oesophageal cancer was associated with social deprivation: patients in areas with the highest levels of socio-economic deprivation had significantly higher death rates than those in areas with lower levels of deprivation. We found a weaker and statistically nonsignificant association between postoperative mortality and deprivation for gastric cancer. This calculation was based on smaller numbers than that for oesophagectomy and hence lacked statistical power. Table 6 shows several factors that may influence the overall health status of patients in areas with socio-economic deprivation.

Although the exact percentage of operations performed for palliative rather than curative reasons was unknown for this data set, a detailed analysis of previous published data (McCulloch *et al*, 2003) has shown that approximately 2.7 and 8.8% of oesophagectomies and gastrectomies for cancer were performed for palliative reasons alone. We did not evaluate the effect of deprivation on operability in this study (although it might be suspected that it may be an important influence, given the tendency for patients from deprived communities to present late – see below) because the nature of HES data meant that we were unable to obtain a reliable denominator for the necessary calculations.

There is considerable evidence that people from lower socio-economic groups have poorer health, with a higher prevalence of common cancers among areas with the greatest levels of deprivation and increased rates of coronary heart disease and smoking-related diseases in the more deprived areas (Carstairs, 1995). The effect of social deprivation on health outcomes has been reviewed on a national level in England for the Department of Health (Black *et al*, 1980; Acheson, 1998). These inequalities translate from birth via increased neonatal morbidity and hospital admissions (Manning *et al*, 2005), to an increase in the incidence of oesophageal cancer among deprived patients who are also heavier smokers and drinkers (Brown *et al*, 2001).

Patients in areas with greater social deprivation may also have inequities in accessing health care resources, and this has been shown, for example, in obtaining a place on the waiting list for organ transplant (Oniscu *et al*, 2003) and in a lower than expected rate of operations for, for example, carotid endarterectomy surgery (Mackenzie *et al*, 2003). It is therefore plausible that patients in areas with high levels of social deprivation might be in a poorer state of health at the time of surgery for gastric or oesophageal cancer compared to patients in areas with little or no social deprivation. The poorer health of these socially deprived patients may then translate into increased postoperative complications and subsequent increased overall mortality. We could also hypothesise that patients in areas of social deprivation might receive less adequate or less timely care, or comply with treatment less well. Interpretation of our data was made more difficult by the apparent difference between oesophagectomy and gastrectomy results. This may simply reflect the smaller numbers in the gastrectomy population, which reduced the power to identify differences between deprivation quintiles. It is also possible that, where gastric and oesophageal cancers are dealt with by different types of surgical departments, differences in the selection of patients for surgery might explain the difference between the two cancers. Units performing oesophagectomies (which were already strongly regionalised by this time) might have been less selective in this respect than those performing only gastrectomies, leading to a gradient in mortality for oesophagectomy attributable to the underlying poorer health status in patients from highly deprived areas.

The influence of important confounding factors known to affect outcome, such as patients' ASA grade and tumour stage, and hospitals' high/low volume status cannot be determined from the HES data used in this study. Further work in a group of patients who undergo oesophagectomy with full information about prognostic factors, including information about prior health status and socio-economic status, would shed light on the probable reasons for the association between deprivation and mortality. Analysis of the effects of likely prognostic confounders (e.g. ASA) is planned, using a suitable comprehensive data set. Measuring access to care and quality of care are more difficult undertakings, and a prospective study might be required to show whether deprived patients were disadvantaged in either regard. The analysis of HES data is only as reliable as the quality of data that are entered into the HES database. All entries into this national database are verified and validated, but a degree of inaccuracy in HES data is recognised. For surgical diagnosis and operation codes HES data appears to be reasonably accurate: a systematic review of 21 studies of accuracy of UK hospital statistics (Campbell *et al*, 2001) illustrated that there was a high level of accuracy for diagnostic codes (91% median) in England and Wales. The accuracy for operation or procedure codes was lower (69.5% median). A comparison of a surgical audit system with routine hospital statistics (Gough *et al*, 1980), reported that there was very close agreement between the two for ‘straightforward procedures’. They reported, however, that there were errors in the detail of more complicated procedures. For inaccuracies in the HES data to introduce bias in our study, it would be necessary for differences in HES data accuracy to exist between lower and higher socio-economic groups. This cannot be ruled out, but there is no evidence for it in contemporary studies and reports. A sample study cross referencing HES data with case records could establish whether there is any cause for concern in this respect, although current privacy concerns may now preclude such a study.

If the effect of social deprivation on oesophagectomy mortality is confirmed after taking into account the known prognostic variables, the implications for care may include the introduction of additional supportive or monitoring care for patients from areas with a high level of social deprivation. If actual differences can be identified between different quintiles in the level of care provided, alterations in the care of all oesophagectomy patients to conform more to the standards provided for the patients in the group with the lowest deprivation (and the lowest mortality) should occur.

We conclude that there is a significant association between social deprivation and mortality after oesophagectomy in current UK practice. Further research is needed to see whether social deprivation is in itself a predictor of a poor outcome after oesophagectomy, or whether it acts as a surrogate measure for other known prognostic variables. The results for oesophagectomy show a significant trend which now needs investigation using individual based data, and this work is currently in progress. The apparent lack of a similar association for mortality after gastric cancer surgery also requires further study.

## Figures and Tables

**Table 1:** Mean age of patients undergoing resectional surgery for gastric or oesophageal cancer by deprivation quintile

	**Average age in years by IMD quintile**				
**Operation**	**1**	**2**	**3**	**4**	**5**
Oesophagectomy	63.3	63.8	64.3	64.5	64.1
Gastrectomy	69.0	69.8	70.6	70.2	70.8

**Table 2:** The 30- and 90-day death rates following operation for oesophageal cancer

		**No. of deaths**		**Standardised death rate per 100 patients**		**95% CI**	
	**No. of admissions**	**30 day**	**90 day**	**30 day**	**90 day**	**30 day**	**90 day**
Quintile 1	1704	162	257	9.87	15.56	8.41–11.52	13.72–17.59
Quintile 2	1325	125	198	9.45	14.93	7.87–11.27	12.93–17.17
Quintile 3	1208	80	142	6.45	11.52	5.12–8.04	9.70–13.58
Quintile 4	1013	78	116	7.48	11.21	5.91–9.33	9.26–13.45
Quintile 5	900	65	108	7.17	11.95	5.53–9.14	9.80–14.43

Overall 30 day CFR=8.3 per 100, test for trend: *χ*^2^=9.5 df=1 *P*=0.002.

Overall 90 day CFR=13.4 per 100, test for trend: *χ*^2^=12.6: df=1: *P*=0.0004.

**Table 3:** The 30- and 90-day death rates following operation for gastric cancer

		**No. of deaths**		**Standardised death rate per 100 patients**		**95% CI**	
	**No. of admissions**	**30 day**	**90 day**	**30 day**	**90 day**	**30 day**	**90 day**
Quintile 1	1739	162	283	9.65	16.79	8.22–11.25	14.89–18.86
Quintile 2	1081	88	151	8.10	13.95	6.50–9.98	11.82–16.37
Quintile 3	832	74	124	8.70	14.54	6.83–10.92	12.09–17.34
Quintile 4	642	60	88	9.21	13.57	7.03–11.86	10.89–16.73
Quintile 5	538	47	86	8.29	15.21	6.09–11.03	12.16–18.79

Overall 30 day CFR=8.9 per 100, test for trend: *χ*^2^=0.6: df=1: *P*=0.46.

Overall 90 day CFR=15.1 per 100, test for trend: *χ*^2^=2.0: df=1: *P*=0.16.

**Table 4:** Odds ratios for deaths in most deprived IMD quintile relative to that in least deprived quintile following each type of operation at 30 and 90 days with their 95% confidence intervals given in brackets

**Operation**	**30 days**	**90 days**
Oesophagectomy	1.37 (1.03–1.85)	1.30 (1.04–1.64)
Gastrectomy	1.16 (0.84–1.62)	1.10 (0.86–1.41)

**Table 5:** CFRs for deaths within 30 and 90 days of oesophagectomy and gastrectomy by IMD quintile (from least to most deprived) for younger and older patients

				**CFRs by IMD quintile (from least to most deprived)**							
**Operation outcome/age in years**	**No. of patients**	**No. of deaths**	**CFR per 100**	**5**	**4**	**3**	**2**	**1**	**IMD trend**	**95% CI**	**Comparison of slopes**
*Oesophagectomy*											
Deaths with 30 days											
<65	2961	148	5.0	4.2	4.6	4.2	5.1	6.0	0.099	−0.017–0.217	
65+	3189	362	11.3	9.9	10.4	8.6	13.8	13.0	0.097	0.025–0.170	*P*=1.0 NS
Deaths with 90 days											
<65	2961	262	8.8	7.7	7.4	7.2	9.3	11.0	0.114	0.027–0.200	
65+	3189	559	17.5	15.9	15.1	15.5	20.6	19.2	0.079	0.023–0.135	*P*=0.9 NS
											
*Gastrectomy*											
Deaths with 30 days											
<65	2063	109	5.3	4.5	4.8	4.9	5.6	5.6	0.046	−0.094–0.186	
65+	2769	322	11.6	11.3	12.6	11.5	9.9	12.6	0.021	−0.055–0.097	*P*=0.7 NS
Deaths with 90 days											
<65	2063	202	9.8	8.0	7.8	10.7	9.2	10.8	0.069	−0.033–0.017	
65+	2769	530	19.1	20.8	17.9	17.6	17.3	21.3	0.028	−0.028–0.085	*P*=0.5 NS

Rates are standardised for age and sex.

**Table 6:** Factors that are potentially associated with increasing levels of socio-economic deprivation for upper GI cancers

Patient-related smoking, alcohol, poor nutrition, obesity, increased incidence of coronary artery disease, patient's overall health status
Local healthcare-related inequities in accessing health care resources, later presentation to a specialist with more advanced disease, longer waiting lists for operations, lower operative rates in deprived areas, perioperative care of patients in high and low volume centres (case volume load of centre and case volume load of surgeon)
Aetiological factors-type of cancer, that is, squamous carcinoma of oesophagus (associated with smoking and alcohol), or adenocarcinoma of the oesophagus or stomach
